# Combined Treatments and Therapies to Cure Spinal Cord Injury

**DOI:** 10.3390/biomedicines12051095

**Published:** 2024-05-15

**Authors:** Nicolas Guérout

**Affiliations:** Saints Pères Paris Institute for the Neurosciences, Université Paris Cité, CNRS UMR8003, 75006 Paris, France; nicolas.guerout@u-paris.fr

Traumatic injuries of the spinal cord (SCIs) are still pathologies with a disastrous outcome. In humans, they most often lead to permanent motor, sensitive, and sphincter deficits, and to this day, there is no available treatment. While a few decades ago, SCI mainly affected young adults, today, it also affects older people in developed countries. The most common causes are road accidents or falls, but they can also be inflicted by knives or firearms [1]. 

Although these injuries have been described as incurable since ancient Egypt, numerous research teams have been interested in developing treatments to repair the spinal cord and thus bring about at least a partial recovery of lost functions [2]. 

In parallel with this research into therapies to improve the management of SCIs, a great deal of work has gone into better describing and understanding the mechanisms that take place after them. Thus, even if there is still some debate within the scientific community, the events that follow the injury and contribute to the development of spinal scars are becoming better understood. Similarly, the cell populations that make up the scar and the role they play have been investigated and are now clearly defined [3]. 

The fundamental knowledge provided by these studies has led to the development of a number of therapeutic avenues, which have been tested after SCIs in recent decades. These include peripheral nerve grafts to bypass the damaged area, the use of enzymes to degrade certain molecules in the extracellular matrix that inhibit axonal regrowth, and the use of antibodies to block the action of inhibitory molecules [4,5,6]. 

However, although these different strategies have shown promising results in animals, particularly rodents, they remain either difficult to implement in humans or have failed to demonstrate their efficacy in clinical trials [7]. 

More recently, the contribution of fundamental knowledge provided by the use of transgenic mouse lines, the latest omics techniques, and optogenetics has enabled us to better characterize cell diversity both in the injured and uninjured spinal cord and in its connections with the cortex and brain stem [8,9,10,11,12,13,14,15]. As a result, research into treatments is focusing on modulating spinal scar and inflammation, inducing the regrowth/survival of the motor and sensitive axons, and promoting new functional connections between the brain and spinal cord [16,17,18]. As a result, a great deal of research is based on cell transplantation, neuromodulation, or physiotherapy [9,19]. 

It is in this context that the project for this Special Issue, entitled “Combined Treatments and Therapies to Cure Spinal Cord Injury”, was designed. The Special Issue includes six research papers and five literature reviews dealing exclusively with cell transplantation, neuromodulation, and physiotherapy techniques.

Indeed, among the six research papers, one concerns the study of inflammation after SCIs, two deal with cell transplantation, and three are related to neuromodulation techniques. 

Similarly, two of the reviews are devoted to cell transplantation, two to new physiotherapy techniques, and one to chronic pain pathologies.

Sabirov et al., in their clinical study, investigated the level of different cytokines present in the blood and cerebrospinal fluid (CSF) at different times post-injury, allowing a characterization of the dynamics of post-SCI inflammation in patients [20].

Kumru et al. studied the effect of transcutaneous spinal cord stimulation (tSCS) applied at the thoracic and cervical level to increase respiratory capacity in SCI patients. They demonstrated that tSCS coupled with inspiratory muscle training (IMT) increases patients’ respiratory capacity, whereas IMT alone does not increase the various parameters measured [21].

Neves Vialle et al. investigated the effects of human mesenchymal stem cell transplantation in a rat model of SCI and demonstrated that, in their model, these cells increased neuronal survival at the site of injury [22].

Georgelou et al. investigated the effects of a combined treatment involving the administration of small-molecule mimetics of endogenous neurotrophins and neural stem cells, demonstrating the synergistic effect of this combination in a mouse model of SCI by means of functional and histological studies [23].

Leszczyńska et al. compared the effects of different neuromodulation methods such as repetitive transcranial magnetic stimulation (rTMS) and peripheral electrotherapy with a physiotherapy method: kinesiotherapy. They particularly demonstrated that the two neuromodulation techniques lead to better patient outcomes than kinesiotherapy alone and that the best results are obtained in the groups combining electrotherapy with kinesiotherapy and rTMS with kinesiotherapy [24].

Tharu et al. compared the effects of a neuromodulation technique, transcutaneous electrical spinal cord stimulation, with a physiotherapy technique, conventional task-specific rehabilitation, in patients with spinal cord injuries, demonstrating that both treatments induce functional recovery [25].

Two reviews in this Special Issue address the contribution of cell transplantation after SCI: Wen et al. provide an overview of the use of dental-derived stem cells, while Reshamwala et al. present various clinical studies that have used olfactory ensheathing cell transplantation in humans [26,27].

Two reviews focus on the effects of rehabilitation in patients with SCIs. Indeed, He et al. present a review of the effects of physical exercise on the reorganization of communication between the brain and the spinal cord, while Stanciu et al. describe the reported effects of rehabilitation through hydrotherapy [28,29].

Finally, the last review by Foreman et al. focuses on lower back pain and clinically presents the various pathologies that can lead to these chronic pains. They also introduce new therapeutic approaches that could help manage these types of pathologies, including neuromodulation and cell transplantation [30].

This Special Issue provides an overview of the latest research on spinal cord injuries, both in animals and in SCI patients. The various published articles highlight promising techniques such as cell transplantation and neuromodulation. They also emphasize the crucial importance of physiotherapy and the various associated rehabilitation methods. These different studies and reviews demonstrate, if it were still necessary, that future treatments must combine various complementary approaches in order to effectively treat this devastating pathology.
